# Andrographolide Inhibits Proliferation and Metastasis of SGC7901 Gastric Cancer Cells

**DOI:** 10.1155/2017/6242103

**Published:** 2017-01-18

**Authors:** Lei Dai, Gang Wang, Wensheng Pan

**Affiliations:** ^1^Department of Gastroenterology, Tongde Hospital of Zhejiang Province, Hangzhou, Zhejiang, China; ^2^Department of Gastroenterology, The Second Affiliated Hospital, Zhejiang University, School of Medicine, Hangzhou, Zhejiang, China; ^3^Tumor Institute of Integrative Medicine, Zhejiang Provincial Academy of Traditional Chinese Medicine, Tongde Hospital of Zhejiang Province, Hangzhou, Zhejiang, China

## Abstract

To explore the mechanisms by which andrographolide inhibits gastric cancer cell proliferation and metastasis, we employed the gastric cell line SGC7901 to investigate the anticancer effects of andrographolide. The cell survival ratio, cell migration and invasion, cell cycle, apoptosis, and matrix metalloproteinase activity were assessed. Moreover, western blotting and real-time PCR were used to examine the protein expression levels and the mRNA expression levels, respectively. The survival ratio of cells decreased with an increasing concentration of andrographolide in a dose-dependent manner. Consistent results were also obtained using an apoptosis assay, as detected by flow cytometry. The cell cycle was blocked at the G2/M2 phase by andrographolide treatment, and the proportion of cells arrested at G1/M was enhanced as the dose increased. Similarly, wound healing and Transwell assays showed reduced migration and invasion of the gastric cancer cells at various concentrations of andrographolide. Andrographolide can inhibit cell proliferation, invasion, and migration, block the cell cycle, and promote apoptosis in SGC7901 cells. The mechanisms may include upregulated expression of Timp-1/2, cyclin B1, p-Cdc2, Bax, and Bik and downregulated expression of MMP-2/9 and antiapoptosis protein Bcl-2.

## 1. Introduction

Gastric cancer (GC) is one of the most prevalent malignancies. Nearly 1 million new diagnoses and approximately 0.74 million deaths occur worldwide [1]. Indeed, GC is the third most common cancer after lung cancer and liver cancer (breast cancer for women) in China [2–4], with 0.3 million new diagnoses and 0.4 new deaths from GC [4], accounting for 40% of GC patients globally [3].

Although surgery is still the first choice for GC, most GC patients are at advanced stages upon the initial diagnosis. Furthermore, patients with advanced GC commonly have distant metastasis or/and local invasion, and 50% of relapsed patients exhibit local lymph node metastasis [3]. Thus, chemo- and radiotherapy (or other means) are the preferred approach for treating advanced GC; common drugs include oxaliplatin, 5-FU, and semustine [4]. Although an increasing number of new drugs are being developed and launched, the prognosis is dismal, with a 5-year survival rate of only 23–36% [5]. Thus, more effective new drugs are urgently needed. Recently, some naturally derived drugs have shown attractive properties, including andrographolide [6].

Andrographolide (ANDR) is a diterpene lactone that is one of the major components of* Andrographis paniculata *Nees, a member of the Acanthaceae family and a commonly used Traditional Chinese Medicine with attributes of clearing away heat and toxic materials, cooling the blood, and reducing swelling [7]. The molecular formula of ANDR is C_20_H_30_O [8]. Several clinical drugs directed at anti-inflammation, anti-infection, immune system regulation, anticardiovascular disease, and anticancer effects have been developed based on ANDR [9–12].

In the present work, we explored the antiproliferation and antimetastatic functions of ANDR and the potential molecular mechanisms involved.

## 2. Materials and Methods

### 2.1. Cell Culture

The GC cell line SGC7901 was purchased from the Shanghai cell bank of the Chinese Academy of Sciences. The cells were cultured in RPMI 1640 medium (HyClone, China) with 10% fetal bovine serum (FBS) (HyClone, China) in a humidified chamber with 5% CO_2_ at 37°C.

### 2.2. Reagents and Kits

A 20 mg sample of ANDR (Sigma, USA) was dissolved in 2 ml DMSO (Sigma, USA) to produce a 10 mg/ml solution. MTT (Sigma, USA), Matrigel (BD, USA), a SYBR Premix ExTaq (Perfect Real Time) kit (TAKARA, China), a cell cycle kit (BD, USA), and an apoptosis kit (BD, USA) were used in this study.

### 2.3. MTT Assay

Cultured cells were trypsinized for the preparation of single-cell suspensions, which were then seeded in 96-well plates at 5000 cells/well. After 12 h, media was replaced with fresh media containing DMSO (DMSO group) or ANDR (ANDR groups). For each group, 3 wells were run in parallel. All media were incubated for 12 h, 24 h, 48 h, and 72 h before the MTT solution was added, followed by 4 h of incubation at 37°C. The supernatant was discarded and 150 *μ*l DMSO was added. After 10 min of shaking, absorption was measured at 490 nm using a microplate reader.

### 2.4. Flow Cytometry for Cell Cycle and Apoptosis Analyses

Cells treated under different conditions for 48 h were detached from 6-well culture plates, washed once with ice-cold PBS, and pelleted by centrifugation. The cells were then suspended in 75% ethanol and incubated on ice for 10 min. The cells were centrifuged at 2500 rpm for 5 min and washed twice with ice-cold PBS. The cell pellets were resuspended in buffer containing PI (Propidium Iodide) and RNase for 15 min in the dark at 37°C, and the cell cycle was examined by flow cytometry after filtration.

For apoptosis, both adherent and suspended cultured cells were harvested. After centrifugation at 2500 rpm for 5 min, the cell pellets were resuspended in binding buffer with FITC-labelled Annexin-V and PI dye and incubated for 20 min on ice. Apoptosis was detected by flow cytometry using a BD Canto II (BD, USA).

### 2.5. Wound Healing Assay

Cells cultured in 6-well plates were scratched with a pipet tip and then washed once with medium. The cells were examined by microscopy at 0 h and 24 h after scratching.

### 2.6. Transwell Assay

Matrigel was diluted with PBS for a final concentration of 50 mg/l, added to the bottom of a transwell, and incubated overnight at 4°C. After discarding the medium, 10^5^ cells were plated into the upper chamber of the transwell (Coring, USA) and cultured with medium containing 2% FBS; medium containing 20% FBS was added to the lower well. After 48 h or 72 h, the cells were fixed with methanol and stained with 0.1% crystal violet. Microscopy (Olympus, Japan) was used to image the attached cells [13].

### 2.7. Western Blotting

Cells in 6-well plates were washed twice with PBS and the supernatant was removed. Cell lysis buffer (50 mmol/l Tris at pH 7.4, 50 mmol/l NaCl, 0.1% Triton X-100, 0.1% SDS, 0.3 mmol/l sodium orthovanadate, 50 mmol/l NaF, 1 mmol/l dithiothreitol, 10 *μ*g/ml leupeptin, and 5 *μ*g/ml aprotinin) [14], RIPA, and the proteinase inhibitor, PMSF, were added to wells. The cells were incubated for 15 min at 4°C or on ice. The cell lysate was centrifuged at 12000 rpm at 4°C. The protein concentration of the supernatant was determined using the BCA kit (Beyotime, China). The supernatant was combined with protein loading buffer and denatured at 100°C for 10 min. SDS-PAGE was performed, and the separated proteins were transferred onto membranes (Millipore, USA). The membranes were incubated with antibodies (Abcam, USA). The ZE-ECL kit (Millipore, USA) was used to visualize the protein bands, and a Bio-Rad instrument (Quantity One) was used for imaging.

### 2.8. Gelatin Zymography

Gel zymography was carried out as previously described [15]. The protein supernatant obtained above was denatured in loading buffer and loaded onto an SDS-PAGE gel containing 1% gelatin. After electrophoresis, the gels were soaked in washing buffer (500 ml 2.5% Triton X-100, 12.5 ml 50 mol/l Tris-HCl (1.5 M), 16.7 ml 5 mol/l CaCl_2_, and 2.5 mL 1 *μ*mol/l ZnCl_2_) for 15 min, repeated 4 times. The gels were transferred and washed twice with washing buffer (50 mol/l Tris-HCl, 5 mol/l CaCl_2_, and 1 *μ*mol/l ZnCl_2_), followed by incubation in buffer (50 mol/l Tris-HCl, 5 mol/l CaCl_2_, 1 *μ*mol/l ZnCl_2_, and 0.02% NaN_3_) for 48 h at 37°C. After the staining and destaining steps, the two apparent bands were scanned using a digital scanner [15].

### 2.9. Real-Time PCR

Total RNA was extracted from cells in 6-well plates using Trizol reagent (Invitrogen, USA) following the manufacturer's recommendations. Complementary DNA was obtained from the RNA using the PrimeScript RT reagent kit (TaKaRa, Dalian), and real-time PCR was performed using a StepOnePlus instrument (ABI, USA) with the SYBR Premix ExTaqII kit (TaKaRa, China). The reaction was cycled at 95°C for 5 min, followed by 40 cycles of 95°C for 5 s, and 60°C for 34 s; the melting curve was obtained from 62°C to 95°C. The primers used for real-time PCR are listed in Table 1.

### 2.10. Statistics

All data were statistically analyzed using SPSS19.0 software. Numerical data are displayed as the mean ± SD, and one-way ANOVA or Student's *t*-test was used to compare differences between different groups. A *P* value of <0.05 was regarded as statistically significant.

## 3. Results

### 3.1. ANDR Inhibited SGC7901 Cell Proliferation

**Figure 1:**
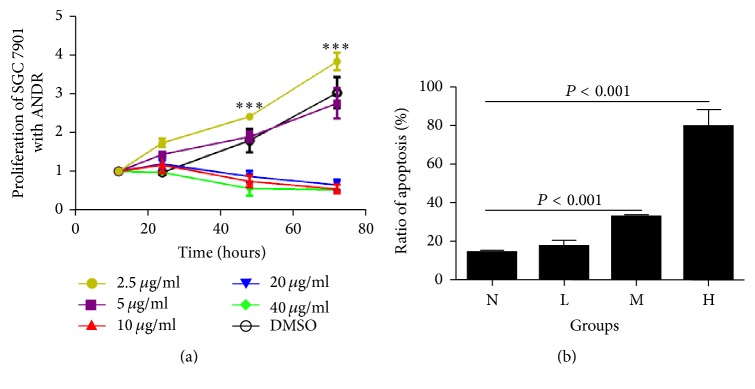
ANDR inhibited proliferation and induced apoptosis in SGC 7901 cells. (a) ANDR at different concentrations inhibited proliferation of SGC 7901 cells. ANDR-treated groups at 40 *μ*g/ml, 20 *μ*g/ml, and 10 *μ*g/ml had a higher capacity of inhibition on the proliferation of gastric cancer cells than the DMSO-treated groups (control) at 5 *μ*g/ml and 2.5 *μ*g/ml (*P* < 0.001). There were no significant differences among the DMSO group treated at 2.5 *μ*g/ml and 5 *μ*g/ml. (b) High dosage (H), middle dosage (M), and low dosage (L) ANDR treatment induced apoptosis of SGC 7901 cells. The ratio of apoptotic cells in H group was higher than that of other groups (*P* < 0.001). The ratio of apoptotic cells in M group was higher than L and N (control) groups (*P* < 0.001). There was no significant difference between N group and L group.  ^*∗∗∗*^After 48 h, the proliferation of groups at 10 *μ*g/mL, 20 *μ*g/mL, and 40 *μ*g/mL of ANDR was inhibited significantly compared to control group (DMSO group) and groups of 2.5 *μ*g/mL and 5 *μ*g/mL of ANDR.

ANDR inhibited SGC7901 cell proliferation at concentrations ranging from 5 to 40 *μ*g/ml, in a time- and dose-dependent manner (Figure 1(a), *P* < 0.01). With this observation, we chose 40 *μ*g/ml, 20 *μ*g/ml, and 5 *μ*g/ml as high (H), moderate (M), and low (L) doses of ANDR in this experiment, respectively. The three doses all significantly decreased the ratio of proliferative cells of SGC7901 cell induced by ANDR compared to the control group (DMSO group) (*P* < 0.01) (Figure 1(b)).

### 3.2. ANDR Blocked the GC Cell Cycle

**Figure 2:**
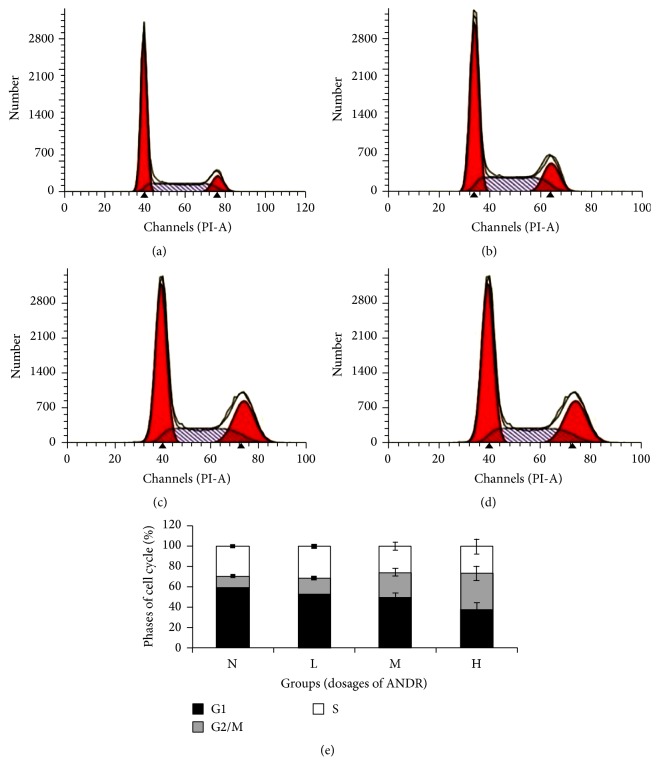
Cell cycle of SGC 7901 cells affected by ANDR at different dosages. (a) Cell cycle of control group (N), (b) cell cycle of low dosage of ANDR group, (c) cell cycle of middle dosage of ANDR group, (d) cell cycle of high dosage of ANDR group, and (e) statistical values of groups at different cell phases. Groups M and H had significantly higher percentages of cells at G2/M phases than groups N and L (*P* < 0.01, Student's *t*-test).

To explore the effect of ANDR on the cell cycle of gastric cancer cells, we examined the cell cycle of SGC 7901 after treatment with different concentrations of ANDR using flow cytometry. SGC7901 cells were incubated with the following concentrations of ANDR for 48 h: 0 *μ*g/ml (N, DMSO group), 40 *μ*g/ml (H), 20 *μ*g/ml (M), and 5 *μ*g/ml (L). Then, analysis of the cell cycle in all groups was performed. As shown in Figure 2, both the high-dose and middle-dose groups of ANDR caused SGC7901 cell cycle arrest at G2/M phase. Meanwhile, the low-dose group and the control group showed 59.01% and 52.32% of SGC 7901 cells in G1 phase, respectively, and increasing concentrations of ANDR decreased the proportion of GC cells at G1 phase (M group 49.84% and H group 37.39%). ANDR at 40 *μ*g/ml and 20 *μ*g/ml arrested the cells at G2/M phase, at 36.2% and 24.39%, respectively. These data suggested that ANDR can block the cell cycle at the G2/M phase (Figure 2).

### 3.3. ANDR Induced Apoptosis in GC Cells

**Figure 3:**
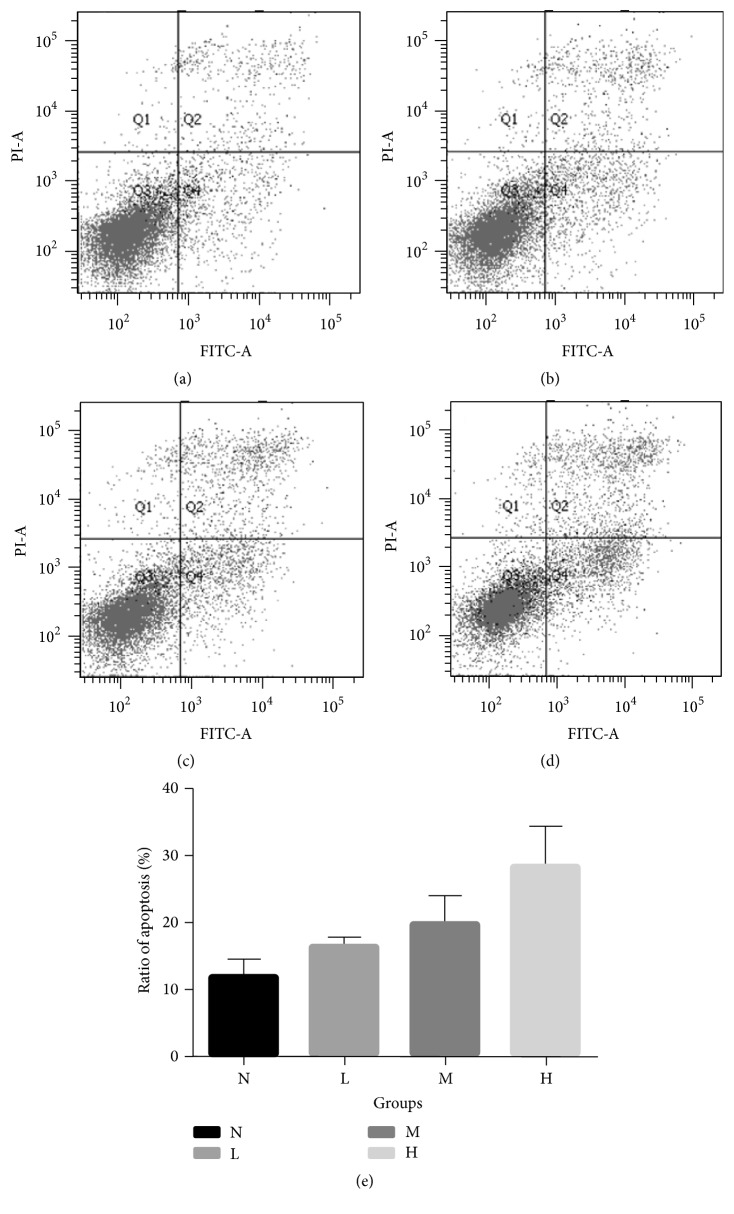
Effects of ANDR with different dosages on GC cell apoptosis. (a) Apoptosis of control group (N), (b) apoptosis of Low dosage of ANDR group, (c) apoptosis of middle dosage of ANDR group, (d) apoptosis of high dosage of ANDR group, and (e) statistical values of apoptosis ratio in different groups. Groups L, M, and H had significantly higher ratio of apoptotic cells than group N (*P* < 0.05 compared to group L, *P* < 0.01 compared to group H and M, using Student's *t*-test).

To confirm the induction of apoptosis by ANDR, media with high, middle, and low dosages of ANDR were used to incubate SGC7901 cells. After incubation for 48 h, apoptosis was examined using flow cytometry with Annexin-V-FITC/PI staining. We found that the apoptosis ratios of the high group, middle group, and low group, were 28.4%, 19.9%, and 16.5%, respectively, and were all higher than the control groups (12%) (*P* < 0.01, compared to group M and H; *P* < 0.05, compared to group L) (Figure 3). The results suggested that ANDR could induce apoptosis of GC cells.

### 3.4. ANDR Decreased Migration Ability

**Figure 4:**
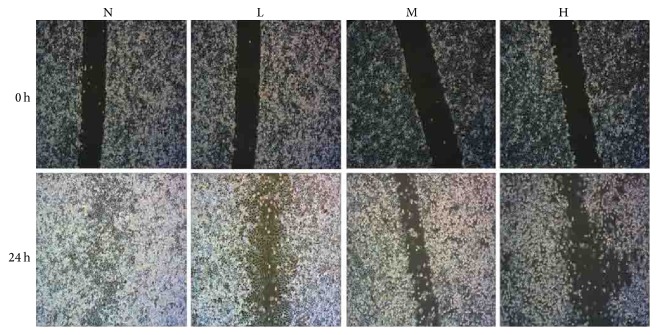
Wound healing of GC cells with ANDR. After incubating with ANDR, GC cells were scratched with pipette tips. After 24 h, cells of the control group had higher migration activity than groups treated by ANDR. In the figure, N denotes control group, H denotes high dosage of ANDR, M denotes middle dosage of ANDR, and L denotes low dosage of ANDR.

To examine whether ADNR influences the migration of GC cells, we performed a wound healing assay. After incubation with ANDR for 24 h, SGC 7901 were scratched and complete media without ANDR was added and the cells were cultured for 24 h. Microscopy imaging of the cells showed greater speed and activity for the cells in the control group compared to the ANDR-treated cells (Figure 4). In addition, increasing ANDR concentrations further decreased the cell migration activity.

### 3.5. ANDR Inhibited GC Cell Invasion

**Figure 5:**
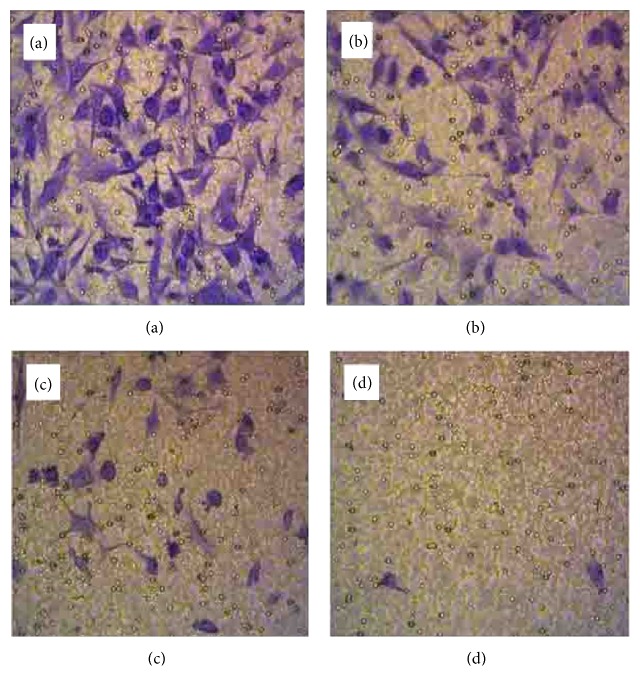
Transwell assay of GC cells treated by ANDR. After incubating with ANDR for 24 h, SGC 7901 cells were seeded into transwell dishes for the invasion assay. As the concentration of ANDR increased, the numbers of transferred cells decreased. (a) Control group, (b) low-dose group, (c) middle-dose group, and (d) high-dose group.

First, SGC 7901 cells were treated by ANDR with different dosages for 24 h. Then, ANDR-treated SGC 7901 cells were detached and plated in transwell dishes for 48 h. After that, the cells were fixed, stained, and imaged. As shown in Figure 5, ANDR decreased the invasion ability of the GC cells in a dose-dependent manner. A reduced number of invasive cells were observed with the high, middle, and low dose groups compared to the control group.

### 3.6. ANDR Reduced MMP-2 and MMP-9 Activities

**Figure 6:**
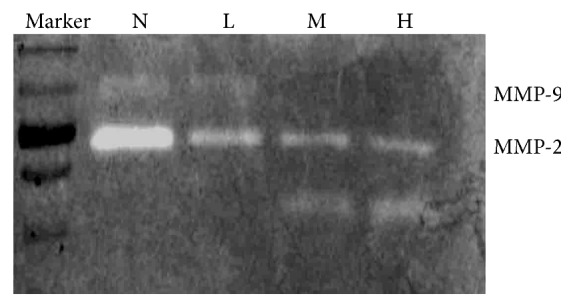
Gelatin-zymography analysis of protease activity of MMP-9 and MMP-2. Gel containing gelatin was used to assess the protease activity of MMP-9 and MMP-2. MMP-9 and MMP-2 activity was decreased as the concentration of ANDR increased. Marker denotes protein marker. In the figure, N denotes control group, H denotes high dosage of ANDR, M denotes middle dosage of ANDR, and L denotes low dosage of ANDR.

Gelatin can be digested by MMP proteins, and decreasing MMP capacity is one of the goals to reduce the incidence of metastasis [16]. Thus, we used a gelatin-containing gel to examine MMP-2 and MMP-9 activities. We found that ANDR affected MMP-2 and MMP-9 activity, compared to the control group. However, no marked difference was found between the different concentrations of ANDR (Figure 6). The data showed that ANDR could reduce protease activity of MMP-9 and MMP-2 secreted from SGC 7901 cells in culture.

### 3.7. ANDR Changed MMP-2, MMP-9, TIMP-1, TIMP2, CD147, Bax, Bik, and Survivin Expression

**Figure 7:**
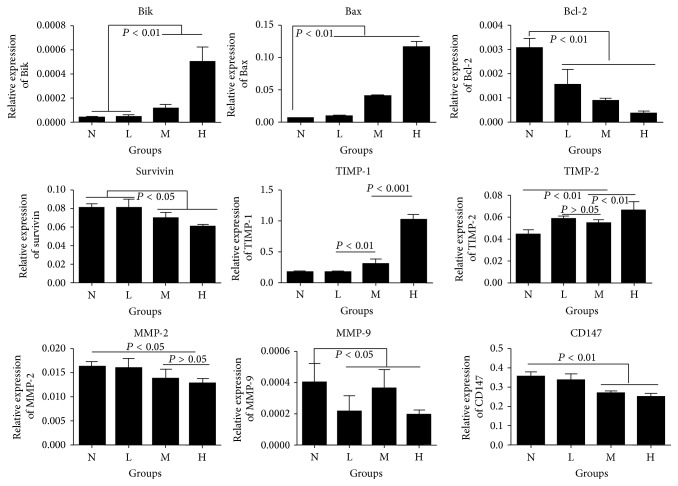
Relative expression of genes associated with invasion and apoptosis of cancer cells. ANDR could have an impact on the expression of genes involved in apoptosis, invasion, and survival. Bik, Bax, and Bcl-2 have roles in apoptosis. Proapoptosis genes, Bik and Bax, were upregulated and antiapoptosis gene, Bcl-2, was downregulated after stimulation by ANDR. Meanwhile, survivin, a gene for sustaining cell survival, was also downregulated after ANDR treatment. TIMP-1, TIMP-2, MMP-2, MMP-9, and CD147 are a cluster of genes involved in cellular invasion, including degradation of extracellular matrix components, digestion of basement membrane, and sustaining cell survival. TIMP-1/2 was significantly upregulated while MMP-2/9 and CD147 was downregulated in GC cells after incubation with ANDR for 48 h.

To explore the potential mechanisms behind the effects of ANDR on GC cells, we performed real-time PCR for the detection of genes involved in apoptosis and invasion of cancer cells. After 48 h of incubation of ANDR and GC cells, real-time PCR was employed to examine the expression of genes with roles in cell invasion (TIMP-1, TIMP-2, MMP-2, MMP-9, and CD147) and in apoptosis and survival (Bcl-2, Bax, Bik, and survivin). TIMP-1/2, Bax, and Bik were upregulated in GC cells, whereas MMP-2, MMP-9, CD147, Bcl-2, and survivin were downregulated. These results indicated that ANDR has the capacity to inhibit invasion and to induce apoptosis (Figure 7).

### 3.8. ANDR Altered Levels of Proteins Related to Metastasis, the Cell Cycle, and Apoptosis

**Figure 8:**
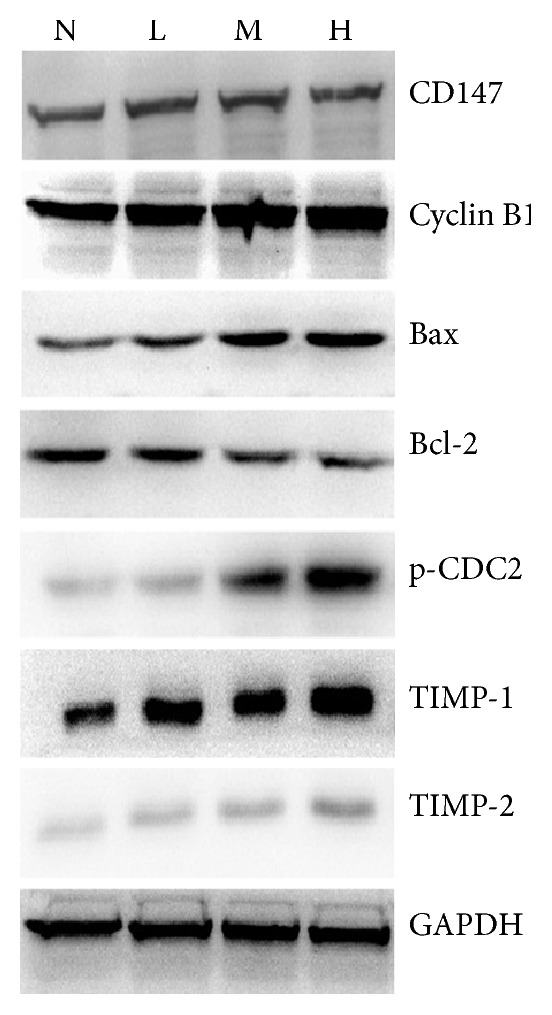
Immunoblots for levels of proteins associated with invasion and apoptosis of cancer cells. GC cells were incubated with ANDR for 48–72 h and cell lysates were collected for western blotting. Apoptosis genes (Bax and Bcl-2), cell cycle genes (cyclin B1 and CDC2), and invasion genes (TIMP-1/2 and CD147) had altered expression levels induced by ANDR at different concentration.

At the mRNA level, we investigated the expression of genes involved in cell invasion, apoptosis, and survival. Next, we examined the protein levels of the same genes. Proteins involved in cancer cell invasion, including TIMP-1, TIMP-2, and CD147, were expressed at increased levels after ANDR treatment (Figure 8). Meanwhile, ANDR could upregulate apoptosis-promoting Bax and downregulate apoptosis-preventing protein Bcl-2 (Figure 8). Additionally, the levels of cell cycle-inhibiting proteins, cyclin B1 and CDC2, were enhanced by with increased concentrations of ANDR (Figure 8). These data suggested that ANDR could suppress proliferation, promote apoptosis, and inhibit invasion by modulating the expression of proteins including cyclin B1, phosphorylated CDC2, TIMP-1, TIMP-2, CD147, and Bcl-2 and Bax.

## 4. Discussion

The high invasion capacity of cancer cells is one of the major reasons for the poor diagnosis of cancer patients. Gastric cancer is a common gastroenterological malignancy, and increasing evidence shows that the invasion and metastasis of this cancer occur in multiple steps and are due to multiple genes with complicated processes. Thus, determining the molecular mechanisms and molecules involved is important for developing highly efficient anticancer drugs. Within this context, an increasing number of herbal drugs are displaying promising effects; andrographolide is one such herb.

In the present work, we found that andrographolide could inhibit the proliferation, invasion, and metastasis of gastric cancer by inhibiting MMP protein activity and upregulating TIMP proteins. Additionally, the expression of apoptosis-associated proteins, such as Bcl-2 and Bax, and some oncogenes, such as survivin, were altered after andrographolide treatment.

Gelatin zymography is used to detect the activity of MMP family proteins. In this study, SGC7901 cells incubated with andrographolide showed decreasing MMP-2 and MMP-9 activities compared with control cells. In addition, the metastasis-associated genes TIMP-1 and TIMP-2 were upregulated by ANDR, whereas CD147 was not.

Inhibiting cancer cell proliferation may be achieved by blocking the cell cycle and preventing invasion and metastasis can be accomplished by altering MMP and TIMP proteins. The degradation of stromal and basal tissues during the migration of cancer cells is a key event for invasion and metastasis. In particular, collagen in the extracellular space may be insensitive to proteases but sensitive to MMPs [17]. To date, there are 28 MMP family members described, including the collagenase subfamily (MMP-1, MMP-8, and MMP-13), the gelatinase subfamily (MMP-2 and MMP-9), the stromelysin subfamily (MMP-3, MMP-10, and MMP-11), membrane-associated MMPs (MMP-14, MMP-15, MMP-16, MMP-17, MMP-23, MMP-24, and MMP-25), and other MMPs, all of which possess a Zn ion-dependent endopeptidase activity homologue domain [18–20].

MMPs have unique roles in tumor progression, invasion, and metastasis. Normally, TIMPs (tissue inhibitors of metalloproteases) act as inhibitors of MMP family proteins, of which there are four members: TIMP-1, TIMP-2, TIMP-3, and TIMP-4. The N-terminus (125 residues) and C-terminus (65 residues) of TIMP contain 3 conserved S-S disulfide bonds [21, 22]. Upregulating TIMPs could restrain invasion and metastasis [23]. There are several steps involved in invasion, but detachment from the basal membrane and ECM degradation by MMPs are key [24]. There are reports that MMP-2 and MMP-9 can degrade ECM components to facilitate invasion [25].

TIMP-1 is an important endoinhibitor of MMP-9, whereas TIMP-2 inhibits MMP-2. TIMP proteins can noncovalently bind to MMP proteins at a ratio of 1 : 1 to inhibit MMP activity in cell invasion and metastasis.

CD147 is a member of an immunoglobulin protein superfamily and is expressed by hematopoietic stem cells and blood cells. The function of the protein is to stimulate fibroblasts and tumor cells to secrete MMPs. Thus, CD147 is an activator of MMPs [26].

## 5. Conclusions

ANDR has antigastric cancer effects in vitro. ANDR may repress the proliferation and metastasis of GC cells by increasing TIMP protein expression and reducing the expression and activities of MMPs. Additionally, the Bcl-2 family may be involved. Further understanding of its anticancer mechanism may result in the broad clinical application of ANDR.

## Figures and Tables

**Table 1:** Primers sequences used in real-time PCR.

Gene		Sequence
MMP2	MMP2-F	TACAGGATCATTGGCTACACACC
	MMP2-R	GGTCACATCGCTCCAGACT
MMP9	MMP9-F	GGGACGCAGACATCGTCATC
	MMP9-R	TCGTCATCGTCGAAATGGGC
BAX	BAX-F	GATGCGTCCACCAAGAAGCT
	BAX-R	CGGCCCCAGTTGAAGTTG
Bcl-2	BCL2-F	TCCGCATCAGGAAGGCTAGA
	BCL2-R	AGGACCAGGCCTCCAAGCT
TIMP1	TIMP1-F	AGAGTGTCTGCGGATACTTCC
	TIMP1-R	CCAACAGTGTAGGTCTTGGTG
TIMP2	TIMP2-F	AAGCGGTCAGTGAGAAGGAAG
	TIMP2-R	GGGGCCGTGTAGATAAACTCTAT
BIK	BIK-F	CTTGATGGAGACCCTCCTGTATG
	BIK-R	AGGGTCCAGGTCCTCTTCAGA
Survivin	Survivin-F	AGGACCACCGCATCTCTACAT
	Survivin-R	AAGTCTGGCTCGTTCTCAGTG
CD147	CD147-R	GAAGTCGTCAGAACACATCAACG
	CD147-R	TTCCGGCGCTTCTCGTAGA
GAPDH	GAPDH-F	AAGGTGAAGGTCGGAGTCAAC
	GAPDH-R	GGGGTCATTGATGGCAACAATA

In the table, the letters F and R stand for forward and reverse, respectively.
